# A Space-Time Analysis of Rural Older People’s Outdoor Mobility and Its Impact on Self-Rated Health: Evidence from a Taiwanese Rural Village

**DOI:** 10.3390/ijerph18115902

**Published:** 2021-05-31

**Authors:** Tzuyuan Stessa Chao, Xiaoqin Jiang, Yi Sun, Jheng-Ze Yu

**Affiliations:** 1 Department of Urban Planning, College of Planning and Design, National Cheng Kung University, Tainan City 70101, Taiwan; tychao@mail.ncku.edu.tw (T.S.C.); p26064059@gs.ncku.edu.tw (J.-Z.Y.); 2 School of Architecture and Civil Engineering, Xiamen University, Xiamen 361005, China; 3 Department of Building and Real Estate, Hong Kong Polytechnic University, Kowloon, Hong Kong; yi.sun@polyu.edu.hk

**Keywords:** space–time path, outdoor mobility, self-rated health, older people, rural, Taiwan

## Abstract

With the aggravation of rural aging, the well-being and self-rated health level of older people in rural communities are significantly lower than those in urban communities. Past studies hold that mobility is essential to the quality of life of the elderly, and well-being depends on their own adaptation strategies in the built environment. Therefore, this study combines three key factors related to active aging: environment, health and mobility, and assumes that the elderly with good health status will have environmental proactivity and a wider range of daily mobility in a poor rural built environment. This study attempts to track daily mobility by using a space–time path method in time geography and then to explore the relationship between outdoor mobility and older people’s self-rated health. A 1-week mobility path survey for 20 senior citizens of Xishi Village, a typical rural village in Taiwan, was conducted by wearing a GPS sports watch. A questionnaire survey and in-depth interviews were done to provide more information about the seniors’ personal backgrounds and lifestyles. The results show that when the built environment is unfit to the needs of daily activities, half of the participants can make adjustment strategies to go beyond the neighborhoods defined by administrative units. Correlation analysis demonstrated that mental health is associated with daily moving time and distance. In addition, men have higher self-rated health scores than women, and there are significant statistical differences between married and widowed seniors in daily outing time and distance. This exploratory study suggests that in future research on rural health and active aging in rural areas, understanding the daily outdoor mobility of the elderly can help to assess their health status and living demands and quickly find out whether there is a lack of rural living services or environmental planning.

## 1. Introduction

A rapidly ageing population is a common phenomenon in the demographic changes of countries all over the world [1]. Taiwanese society is about to face the challenge of a rapidly increasing elderly population in the near future, and the percentage of people with disabilities and dementia will also grow significantly as many other countries [2].

**Figure 1:**
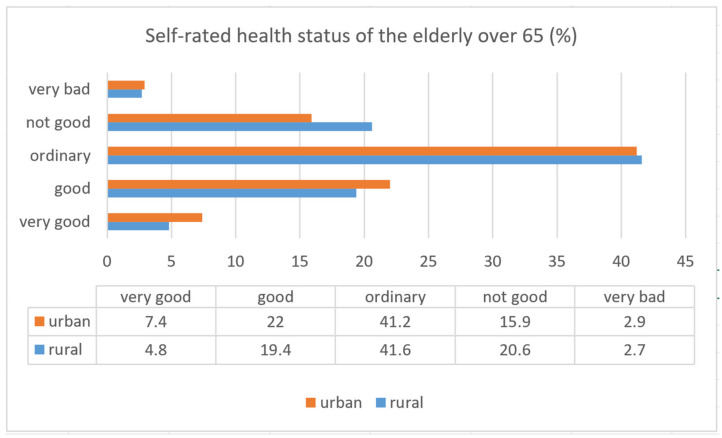
The self-rated health status of the elderly over 65.

Due to the trend of urbanization, young people living in rural areas tend to move to cities to look for job opportunities [3,4], which has led to a higher proportion of older people in rural areas. Compared with the urban population, rural residents have less access to services and activities because of poor socio-economic conditions. Therefore, the rural elderly may face greater risks of social isolation, reduced mobility, lack of support and insufficient medical care [5]. It is reported that rural elders have a higher level of stress and lower levels of well-being and quality of life than urban elders [6,7,8]. According to the latest National Health Interview Report (2013) from the Ministry of Health and Welfare of Taiwan [9], the average self-rated health of rural older residents is significantly worse than that of urban elderly (Figure 1), especially in relation to anxiety and depression. Therefore, it is particularly important to study the health status of rural elders.

A large number of studies also found that daily mobility is an important predictor for older people’s health and well-being, and can enable older adults to maintain their physical health, independence and participation in society [10,11,12,13,14]. In particular, outdoor mobility is strongly correlated with older people’s health status, which means older people with a positive perception of their health status participate in more varieties of daily activities [15]. Therefore, it may assess the health of the elderly by tracking their outdoor mobility.

In addition, older people’s health is affected by the built environment and also influences mobility [16]. If older people want to increase outdoor mobility or daily activities, additional strategies are needed to create an age-friendly environment [17]. An age-supportive environment can foster mobility in older adults [18,19]. However, in a rural built environment, there is a long-term urban-rural gap in the allocation of facilities supporting daily activities [20], which challenges the quality of life of the elderly in rural areas. The distribution of resources directly affects the initiation of different activities. For example, inconvenient and unavailable public transportation limits the ability of elderly people to go out alone [21]; insufficient leisure service resources in rural areas affect the diversity of leisure activities and successful aging of the elderly [22]. Facing the unfriendly built environment, it is worth considering whether the daily mobility and health of the rural elderly are affected by it.

Ageing successfully also is determined by how effectively older people cope with adversity [23]. Older people are not always passive recipients of environmental demands. They can develop “environmental proactivity,” a state with an increased personal capability to make use of resources [24]. Accordingly, the daily outdoor mobility of the elderly needs to respond to environmental challenges (inconvenient transportation or inadequate public facilities) in order to participate in activities needed for daily life and personal welfare (such as shopping or leisure activities) [25]. Based on the interaction of the environment, the health and daily mobility, this study assumes that the elderly with good self-rated health will make adjustment strategies and reach a wider range of daily mobility in the poor rural built environment. A method of space–time path in time geography is used to track their daily mobility in a typical rural village in Taiwan and explores the relationship between their mobility and their self-rated health. Using the GPS tracking method, ‘mobility’ includes the distance covered, the spatial area, and time. In addition, semi-structured interviews are conducted to understand the personal background and activity habits of the elderly. These interviews allow further analysis of the relationship between outdoor mobility and self-rated health and the interaction between older people’s outdoor activities and the neighborhood environment. The findings are expected to feed back to suggested amendments of relevant policy tools and as a reference for the planning and design decisions around the allocation of resources to elderly-friendly villages in the future.

## 2. Literature Review

### 2.1. Rural Built Environment and Older People

In rural areas, the proportion of the elderly is increasing, while the young are steadily moving into cities [26,27]. As a result, the rural population is decreasing, aging and becoming increasingly multi-morbid [28]. However, due to the long-term urban-rural gap in the allocation of supporting facilities for daily activities, the built environment in rural areas challenges the quality of life of the elderly [20,29]. Older people have difficulties accessing shops, banks, health or education and enjoying leisure activities [30].

The built environment is defined as all buildings, spaces, and amenities that are created or modified by people [31]. Theoretically, a good built environment can evoke physical activities and environmental barrier conditions that limit outdoor mobility, especially in older adults [32,33,34]. The interactions between older adults and their environment change with physical and mental health. For example, older adults are unable to participate as much as they wish, often due to mobility constraints [35]. However, in the face of environmental pressure, people can make different behaviors and strategies, including environmental proactivity and reactivity [36]. The well-being of the elderly depends on their positive adjustment strategies to the environment [24,37,38,39,40]. Unlike urban counterparts, rural residents generally have a self-reliant existence and are often able to care for themselves and their families for periods of time without assistance [35]. The rural elderly may be more active in coping with environmental barriers. As a result, environment, personal health and outdoor mobility are closely linked, which are worthy of attention in the study of aging and health.

### 2.2. Older People’s Mobility and Its Association with Self-Rated Health

Maintenance of mobility is fundamental to active ageing, allowing older adults to lead dynamic and independent lives [11,41,42,43]. Low mobility might be an undesired effect of ageing, which reduces participation in out-of-home activities, which, in turn, might also decrease quality of life [44,45]. Mobility is defined as the ability to move oneself within environments that expand from one’s home to the neighborhood and to regions beyond [46]. Mobility includes movement outdoors and indoors. This article focuses on observations of outdoor mobility. Out-of-home mobility is necessary for accessing commodities, making use of neighborhood facilities, and participating in meaningful social, cultural, and physical activities [34]. In rural areas, the purpose of outdoor mobility for seniors may include sports, handling chores, working in the field, hospitals or clinics, leisure and religious activities, visiting relatives or friends, and dining out. By observing the distance, time and scope of the elderly’s daily outing activities, it can better understand their interaction with the built environment. For example, older people who are able to travel greater distances have a higher possibility of engaging in activities outside the home and maintaining shopping and leisure trips [47].

Self-rated health (SRH) is proved to be an effective and valid global measurement tool for poor health and physical function [48,49]. SRH is an individual’s own perception of their health, which involves a large scope of health issues related to social, economic, behavioral, and psychological factors [50,51]. Physical and psychosocial factors play a role in maintaining mobility in old age [52,53,54]. It is reported that the frequency of going outdoors is directly linked to levels of physical activity among community-dwelling older people, and practicing moderate physical activity could amortize its negative effects on psychological health [55]. Additionally, the increase of regular leisure time physical activity accounted for improved SRH [56,57,58,59,60]. The more restricted activities in daily life, the worse mental health, and the lower self-rated health score [61]. Consequently, there is a strong positive association between outdoor mobility and SRH, especially in mental health. However, previous studies have focused more on the physical obstacles of the elderly themselves affecting outdoor activities. In this study, our focus is on the association between outdoors mobility patterns (in terms of time, distance, and area) with physical health and mental health and the interaction between older people’s outdoor activities and the neighborhood-built environment.

### 2.3. Space-Time Path for Understanding Mobility

In the analytical framework of time geography, one of the central concepts is the space–time path, which is used to depict an individual’s trajectory over space and time [62]. The space–time path traces the individual’s physical movement in space with respect to time and highlights the constraining effects of a person’s need to be at different locations at different times [63]. Participating in activities requires trading time for space to access these locations at their available times. The space–time path highlights these requirements and focuses on physical mobility and interaction [64].

It has been recognized that the space–time method is value to help change the way of thinking from static residential spaces to other relevant places and times in people’s everyday lives, and as a result, there have been increasing attempts to address the dimensions of time and mobility in the research and practice of policies and planning [65]. A number of studies have extended, analyzed, and implemented space–time paths to analyze human activity patterns, including mobility related to health and aging. For example, a study assessment of the mental health and mood by tracking the daily mobility path beyond the residential environment [66]. Intelligent devices, especially the global positioning system (GPS), greatly promote the further development of space–time research. GPS data can be used as input data to calculate mobility indicators that describe an individual’s daily mobility patterns [67]. Because of its convenience and controllability, more and more scholars rely on it to capture the outdoor activities of the elderly [67,68,69,70].

However, previous studies applying the space–time approach to health and personal activities mostly focus on the areas and objects of the city [71,72,73,74], less countryside. This study highlights the extent of the space–time path to analyze the scope, time, and distance of outdoor mobility path of rural elderly.

## 3. Methods

**Figure 2:**
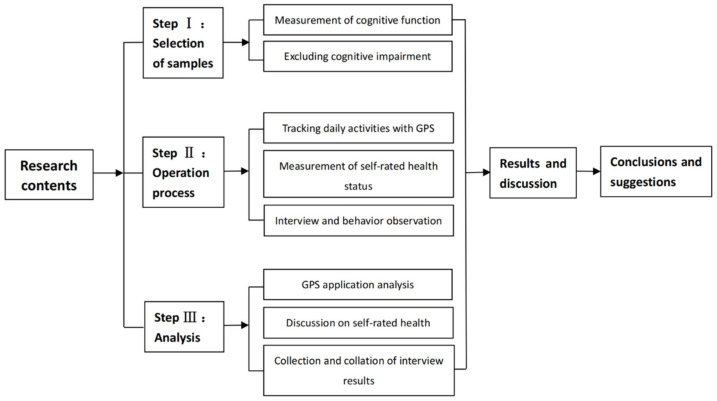
Research Procedures.

In this study, elderly individuals with normal cognitive function were selected as the research objects in a typical rural community, and then each participant was asked to wear a GPS sports watch to locate and record their daily life space–time path in a week. Through the questionnaire and SF-36 scale to understand their basic personal information and self-rated health status, and then through in-depth interviews and behavior observation to further understand their daily activities information, including activity time, activity content, and activity environment, etc., and finally conduct a comprehensive analysis to explore their correlation and influencing reasons. Figure 2 demonstrates the steps of the study.

### 3.1. Location and Participants

**Figure 3:**
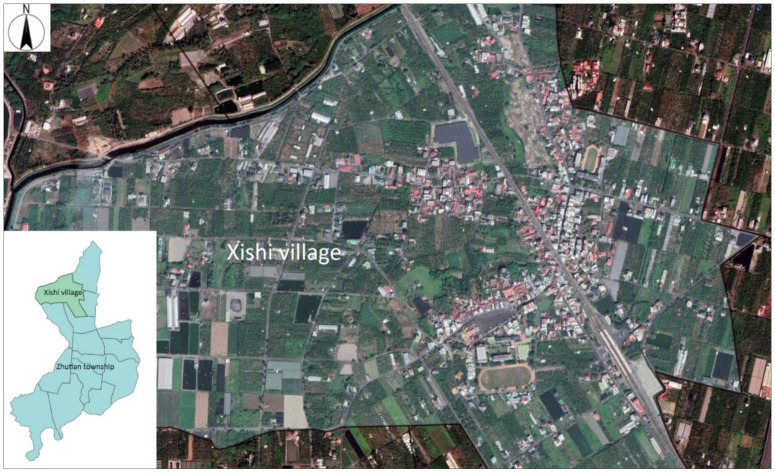
Survey site: Xishi village in the Zhutian township.

This study focused on community-dwelling, ambulatory older people living in Xishi. Xishi (Figure 3) is a village in the Zhutian township, Pingtung County, Southern Taiwan. The topography of Xishi village is flat, which is part of Pingtung plain, and the temperature is higher in summer. In August 2018, the population was 2796. The number of older people (aged 65 and above) was 547, accounting for 19.59% of the total population. Most areas are covered by farmland, and the main industry of Xishi village is agriculture. In terms of economy, the output value from agriculture is lower than that of neighboring villages. The Xishi villagers need to visit neighboring villages or the township to purchase necessities due to the lack of retail shops.

Participants were recruited from older adults aged 55 and above, residing in Xishi village for more than two months. The selection criteria included older people, who speak Chinese or Taiwanese, and no cognitive malfunction (screened by *Short Portable Mental Status Questionnaire,* recruiting participants who did not answer the wrong questions). Finally, 20 participants were recruited.

### 3.2. Measure Tools

The research methods include questionnaires, GPS tracking, and in-depth interviews. The questionnaires are divided into three parts (Files S1–S3). In the first part, the participants are selected by a short portable mental status *Questionnaire (SPMSQ).* In the second part, the basic background information is obtained. Finally, the self-rated health of the participants is measured by the SF-36 scale (Medical Outcomes Study 36-item Short-Form).

#### 3.2.1. Basic Demographic Characteristics Data Sheet

The basic data sheet consists of 10 questions, including gender, age, education level, occupation, religious belief, source of daily living expenses, marital status, housing type, cohabitation status, common transportation mode, etc.

#### 3.2.2. Short Portable Mental Status Questionnaire (SPMSQ)

*SPMSQ* has been proven to successfully replace the long and typical test cognitive function, which can save a lot of time and has been widely used in cognition. The questionnaire consists of ten questions, which of the content includes the sense of orientation of the surrounding affairs, attention of the current event, thinking ability, short-term and long-term memory, sequential mathematical calculation, and general knowledge. Each answer to a right question can be scored one point, with a total score of 10 points, a minimum of 0, and a maximum of 10. A higher score indicates better cognitive function.

#### 3.2.3. GPS Tracking

Each participant was given a GPS watch and asked to wear it for 7 consecutive days. A trained postgraduate research helper visited the participants every morning to make sure the tracker was properly worn and charged. After the GPS locator is properly worn, the respondents’ mobility distance from point A to point B is used for positioning, and the positioning points are automatically stored and recorded. Through these numerous recorded “points,” a “line” is formed, and then an effective mobility path is formed, and the coordinate system data of satellite positioning will be returned. GPS trajectory data can be extracted by GOLiFE CONNECT software, which presents the movement paths for 7 days with a set of locations. By using GPS positioning coordinates in the Google Earth system, the space and image will be combined to better understand the circle of mobility of the elderly.

#### 3.2.4. The Medical Outcomes Study 36-Item Short-Form (SF-36)

The SF-36 was designed as an indicator to evaluate the cognitive function of participants. It includes one multi-item scales measuring eight health concepts: (1) PF (Physical functioning), (2) Role limitations due to physical health problems (RP), (3) Bodily pain (BP), (4) General health perceptions (GH), (5) Vitality, energy or fatigue (VT), (6) Social functioning (SF), (7) Role limitations due to emotional problems (RE), and (8) General mental health, covering psychological distress and well-being (MH). SF-36 items and scales are scored so that a higher score indicates a better health status [75,76].

The scoring steps of SF-36 are as follows: the first step is to code the scale items, and then to score the scale items on each of the eight scales, and finally, these are linearly transformed to a 0-to-100 scale. The formula is:

$$\mathrm{Transformed}\;\mathrm{scale}=\frac{\left(\mathrm{Actual}\;\mathrm{score}-\mathrm{lowest}\;\mathrm{possible}\;\mathrm{score}\right)}{\mathrm{Possible}\;\mathrm{Raw}\;\mathrm{Score}\;\mathrm{Range}}\times 100$$

Due to its shortness and good reliability and validity, there are many versions (including Germany, France, Italy, Japan, the Netherlands, Belgium, Denmark, etc.), and the US original version further develops SF-12. This study refers to IQOLA SF-36 Taiwan Standard Version 1.0.

#### 3.2.5. In-Depth Interviews

One week after participants had worn the GPS device, each one was interviewed. During the interviews, the participants’ mobility practices were discussed in relation to their self-reported health. The content includes: time point, time amount, place, interactive behavior, use of facilities, etc., so as to understand the environment of daily mobility of the elderly and the details of their activities. The interviews were conducted in the homes of the participants, audio-recorded, and transcribed verbatim. Each interview lasted around thirty minutes.

### 3.3. Variables

#### 3.3.1. Dependent Variable: Self-Rated Health (SRH)

The physical component summary (PCS) and mental component summary (MCS) scales summarize the eight SF-36 scale scores into two summary scores that give an overall assessment of the quality of life related to physical and mental health, respectively [77,78]. In this study, two variables of PCS and MCS are used to reflect the general self-rated health status of rural elderly, respectively. The PCS has four items (physical functioning, role limitation due to physical problems, bodily pain, and general health), and the MCS has four other items (vitality, social functioning, role limitation due to emotional problems, and mental health). Summed scores of the respective items will be obtained for analysis. To produce the PCS and MCS summary scores, the z-scores for each of the eight scales are multiplied by a factor score coefficient, and the resulting scores are summed over the eight subscales [75,79]. The SF-36 PCS and MCS scoring algorithm is summarized below:

PCS = ∑ (z-score of each scale × respective physical factor coefficient) × 10 + 50

MCS = ∑ (z-score of each scale × respective mental factor coefficient) × 10 + 50

#### 3.3.2. Independent Variables: Distance, Time, and Space of Mobility

For analysis, ‘time’ refers to the respondent’s total hours spent outside their homes for 7 days, including going out for exercise, visiting children, shopping, etc. The average daily outing time was used for analysis. ‘Distance’ refers to the respondent’s total length of all tracks away from home, and an average daily moving distance was calculated by GPS and used for analysis. ‘Space’ refers to the range of the respondent’s activity space by calculating the standard deviation of their ellipse area [80,81,82,83]. The ellipse area provides a general indicator of the span of the respondents’ activity spaces. The center of the ellipse was drawn based on the mean center of respondent’s observed locations. The standard deviation of x-coordinate and y-coordinate is calculated from the average center as the starting point so as to define the axis of the ellipse. By mapping daily trips and locations of various activities in 7 days, the standard deviation ellipse area was calculated based on the distance and direction of these locations from home. Each respondent’s ellipse and its total area in square miles was calculated in QGIS. An average daily standard deviation elliptical area was used for analysis.

### 3.4. Analysis

Pearson correlation analysis was used to infer the correlations between dependent variables and independent variables. Additionally, an independent-samples *t*-test was used to discuss whether there is a significant difference between metric variables and categorical variables. Explanations from observations and interviews were used to supplement the quantitative analysis in order to understand environmental and some psycho-social factors leading to different movement patterns. The study data was collected and analyzed in SPSS version 17.0 software.

## 4. Results and Discussion

Table 1, Table 2 and Table 3 list the sample characteristics, health conditions, and daily activity types of the participants. The majority of the participants (n = 20) were female (13, 65.0%). The average age was 67.50 (SD ± 5.80) years old. PCS of them (n = 20) was 74.94 ± 12.99, and the MCS was 77.30 ± 9.51. The self-rated health score (PCS and MCS) for the males is higher than that of the females, which is consistent with the previous studies [84,85,86,87]. The daily average moving distance is about 10.84 km, and the daily average travel time is 4.77 h.

### 4.1. Space-Time Path of Older People and Health

**Figure 4:**
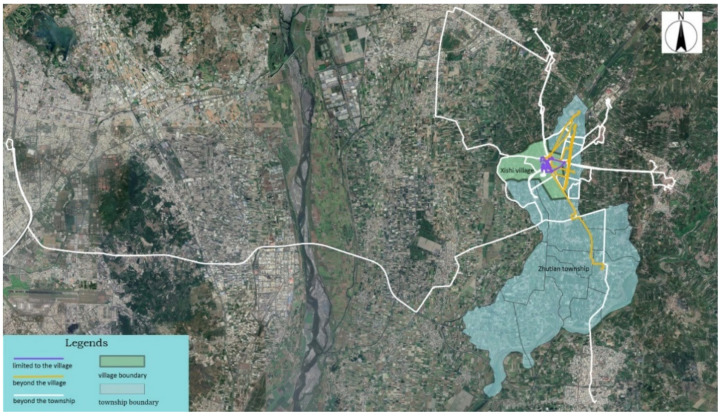
Three categories of space–time paths based on administrative boundaries.

**Figure 5:**
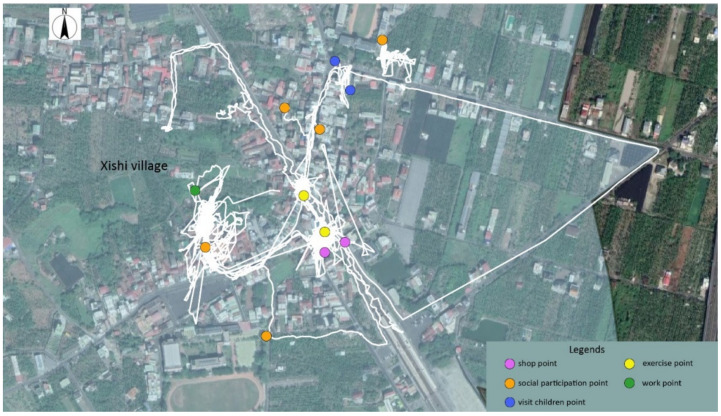
The daily activity locations in one representative day of Category I.

**Figure 6:**
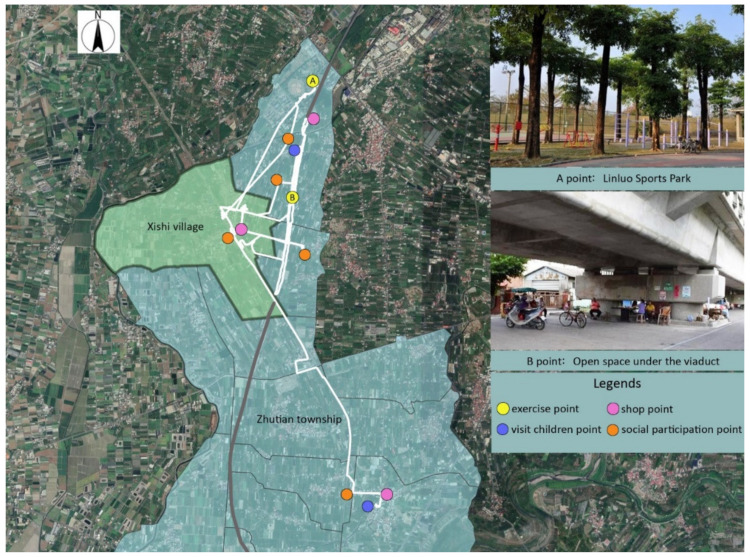
The daily activity locations in one representative day of Category II.

**Figure 7:**
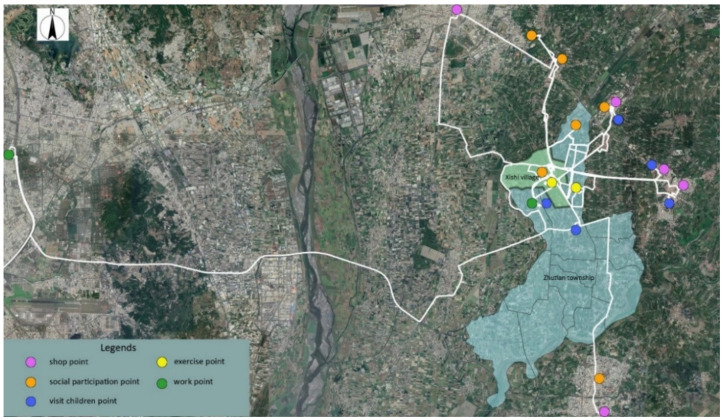
The daily activity locations in one representative day of Category III.

The trajectory of individuals in terms of administrative boundaries was categorized into three groups (Figure 4): (I) limited to the Xishi village, (II) beyond the Xishi village and limited to Zhutian township, and (III) beyond the Zhutian township. Table 4 provides details regarding the physical activities and health among different categories. After in-depth interviews and Participant observations, we can learn more about the location, reasons, and travel methods of their daily activities during the week. Common daily activities for participants include: shopping, exercising, socializing, visiting children, and working. Figure 5, Figure 6 and Figure 7 list the main points of daily activities in one representative day for three categories of participants.

For participants within category I, the average daily standard deviation elliptical area was 0.94 square kilometers, with an average moving distance of 2.90 km. The average daily outing time was 2.37 h. Most of the daily mobility of the elderly of this category is located in Xishi village. Figure 5 indicates they choose to shop, socialize, and exercise occasionally in nearby places. After interviews, some of these elderly people feel that their physical function is not good and their personal abilities are reduced, which limits the scope of daily movement and leads to their low self-rated health scores. In addition, some older people do not have time to go out to take care of their sick spouses and usually only go to nearby mobile vendors to buy some vegetables and daily necessities. Their behavior is affected by the inadequacy of environmental facilities and obeying the environment, and they only maintain daily needs through short commutes.

“Every day, I will go to a mobile vendor to buy fresh vegetables or daily necessities. After my first recovery from a serious illness, my mental health was greatly affected by my husband’s physical disability. I often felt depressed, lost and unable to lift my spirits. After the introduction of my neighbors, I would take time for volunteer service or to have a meal with other elders in the community development association within a week, hoping to improve my mental state.”

Within category II, the average daily standard deviation elliptical area was 4.25 square kilometers. The average daily moving distance was 6.19 km, and the average daily outing time was 3.53 h. Many of the comments we have observed reflect that the elderly do not have enough open space for physical exercise and social interactions. Some of them will choose to travel long distances and find suitable places to carry out autonomous exercise (for example, to walk and run). The most popular locations we collected are the open spaces under one upheaved highway linking north to south in Taiwan and Pingtung Sports Park in the north of Zhutian township (Figure 6). Another part of the elderly is reluctant to go out to exercise and socialize because of the long distance.

“I like to exercise under the viaduct, because it’s convenient to go there, and it’s shade, not afraid of the sun and rain, and the space is long enough for walking. My friends also like to exercise there. Every day we can meet and chat there.”

“At present, the circular footpath and integrated park are under construction in Zhutian middle school, hoping to meet the lack of sports and rest space in the village. After all, we are all older. Every day, when we think of cycling for a distance before we can get under the viaduct, we sometimes feel too lazy to do it.”

Within category III (Figure 7), the average daily standard deviation elliptical area was 17.27 square kilometers. The average daily moving distance was 17.48 km, and the average daily outing time was 6.7 h. There are two reasons behind their long commute beyond the township. First, Xishi village is located in the north of Zhutian township, which is a bit remote with insufficient community facilities such as a market. Many important facilities are located in the adjacent townships as those towns have a larger population and more financial support from the Pingtung government. For example, one villager commented, “there is no traditional market or fresh supermarket in the village... which is very inconvenient to obtain daily necessities.” The senior villagers need to spend more transportation time to obtain basic living resources. The second reason is emigration; the younger generation moves out to search for better employment opportunities or better education services. Most of the children of the older people live in the nearby townships, and the elderly go far to visit their children.

“There is no traditional market or fresh supermarket in the village. I don’t think it’s convenient to buy vegetables and daily necessities. Many fresh fruits and vegetables need to be bought in the markets of Neipu Township in the East, Chaozhou Town in the South and Pingtung city in the north. I need to travel a long distance to these places, so it is often my son’s daughter-in-law who lives in a nearby village who takes me to the market to buy them together, and then carries the grandson who has just finished class back to my son’s home to cook and eat dinner together.”

“My son lives in the next village. He moved out when he was 16. At that time, the next village opened a new factory and the salaries are very attractive. That’s why he decided to leave, although he did not wish to... Now I often go to their home to help them cook and look after their children.”

From the above three categories, we find that older people who present different daily mobility paths have different health perception scores. For Category I, the PCS and MCS were the lowest (62.58 and 70.85, respectively) compared to other groups. Category III travelling beyond the Zhutian township had the highest physical and mental health scores (80.78 and 81.38). Similarly, different daily mobility paths reflect their different adaptation strategies to the environment. The pressure of the rural environment is the key problem they reflect, such as insufficient space for exercise, no space to provide social interaction, lack of traditional markets, and insufficient educational resources lead to a long distance from children, etc. Facing environmental pressure, the older people of Category I feel that they are in poor health, are unwilling to travel, and are more restricted by the environment. Some of Category II choose to self-regulate to respond to environmental influences, such as finding suitable space to daily exercise. Category III feel well in their health and are proactive to travel farther to take care of the children, purchase daily necessities in the next town, and even work farther away.

### 4.2. The Relationship between Mobility and Self-Rated Health

Table 5 uses Pearson correlation analysis to assess if the mobility patterns (in terms of daily moving time, distance, and ellipse area) are associated with the PCS or MCS. We assume that they are correlated, so we apply the Bonferroni correction to the *p*-value, *p* = 0.05/3 = 0.017. We found the MCS is associated with daily moving time (*r_s_* = 0.538, *p* = 0.014) and distance (*r_s_* = 0.556, *p* = 0.011). The results of this study are consistent with the previous literature [55,60]. However, the PCS does not show a correlation in the three aspects of mobility.

Furthermore, we did the correlation analysis between age, education and PCs or MCS in Table 6, and the results indicated that there was no significant correlation between them. However, there is a negative correlation between age and education level (*r_s_* = 0.619, *p* = 0.004). This result shows that the older the elderly in rural areas, the lower the level of education.

There were 7 male and 13 female participants. An independent-samples *t*-test (Table 7 and Table 8) was run to determine if there were differences in self-rated health, including PCS and MCS, between males and females. There were no outliers in the data, as assessed by inspection of a boxplot. PCS and MCS scores for each level of gender were normally distributed, as assessed by a Shapiro–Wilk’s test (*p* > 0.05), but the assumption of homogeneity of variances was violated, as assessed by Levene’s test for equality of variances (*p* = 0.004, *p* = 0.001). Male PCS score (*M* = 86.71, *SD* = 1.89) was higher than female PCS score (*M* = 68.85, *SD* = 12.02). A statistically significant difference, *t* = 5.241, *p* = 0.000. Male MCS score (*M* = 84.71, *SD* = 2.81) was also higher than female MCS score (*M* = 73.15, *SD* = 9.37). A statistically significant difference, *t* = 4.117, *p* = 0.001. The results of this study are consistent with the previous literature: females tend to report lower health status than men in surveys [88].

Furthermore, an independent-samples *t*-test was run to determine if there were differences in outdoor mobility between married and widowed participants (Table 9 and Table 10). There were 16 married and 4 widowed participants. The result showed that there are statistically significant differences in the average daily moving distance (*t* = 2.708, *p* = 0.015) and average daily outing time (*t* = 2.211, *p* = 0.040). However, there is not a statistically significant difference in the average daily standard deviation elliptical area (*t* = 1.328, *p* = 0.201). The moving distance was greater in married participants (*M* = 12.69, *SD* = 11.95) than widowed participants (*M* = 3.48, *SD* = 3.25). Similarly, married participants spent more time going out (*M* = 5.36, *SD* = 2.63) than widowed participants (*M* = 2.38, *SD* = 0.76).

## 5. Conclusions

The novelty of this study is to combine the three factors of active aging, including environment, health and mobility, to reveal the relationship between daily outdoor activities and self-rated health of the rural elderly by using the space–time path method and to analyze the causes of mobility and the possible environmental factors. Based on the results of the GPS and SF-36 questionnaire survey, it shows that outdoor mobility is related to self-rated health. Facing the limitation of the rural built environment, such as the lack of public facilities and convenient transportation, older people with good SRH will select proactive behaviors to adapt existing environment; their daily mobility often extends beyond the local neighborhood. In contrast, the elderly who perceive themselves with poor health are restricted by their living environment, reducing outdoor activities, or making nearby travel. This study has a good value for evaluating the difference in the person–environment fit among older people with different health statuses.

Moreover, many studies have shown that the higher the mobility of living space, the better the quality of life of the elderly [54,89]. Using GPS technology to assess living space mobility has been applied in the research of promoting active aging in various countries [90,91]. This article is a supplement to the research on living space. Motivated by the concern for the health of the rural elderly, this study tracks the trajectory and scope of their daily outdoor mobility, the results of which provide a certain degree of guidance on quickly finding and solving the current rural planning problems and optimizing the structure of rural living space.

Nevertheless, compared with the urban elderly, the respondents in rural areas are generally less educated. As the GPS watch is relatively complicated for them to use, the participants had concerns about using the device independently. Although researchers assisted them to practice many times before the experiment, few of them were able to complete this research. Therefore, the accuracy of the results might be affected by the limited number of observed samples. In future research, we will expand the sample size and do more correlation and regression quantity analysis between mobility and health in terms of time, distance, and scope.

In addition, as a cross-sectional study, this survey was conducted in a single community within a two-month period (from March to April). Therefore, the accuracy of the results may be affected by the climate characteristics, local culture, living habits and other factors. For future research, other convenient tracking tools, including mobile phone data and smart insole tracking, can be used to compare the impact of living space mobility and health perception in different communities and different age groups, which aim to reconstruct compact, convenient and complete rural daily life space, and realize the balanced allocation between residents’ behavior demand and rural public resource supply.

## Figures and Tables

**Table 1:** Narrative statistical analysis of individual background data (N = 20).

Gender	65% Female
Age	Mean: 67.5 years, SD: 5.8 years Range: 55–74
Marital status	Married: 80% Widowed: 20%
Highest level of education attained	Primary: 20%, Secondary school: 20%, High school: 60%,
Means of transport	Bicycle: 5% Motorcycle: 70% Automobile: 25%

**Table 2:** Narrative statistical analysis of self-rated health status (N = 20).

Statistical Value	Mean ± Standard Deviation	Mean ± Standard Deviation (Male)	Mean ± Standard Deviation (Female)
physical functioning, PF	79.75 ± 14.09	86.43 ± 7.48	90.71 ± 9.32
role-physical, RP	68.75 ± 17.91	75.00 ± 0.00	65.38 ± 21.74
bodily pain, BP	80.00 ± 17.66	94.00 ± 0.00	72.46 ± 17.84
general health, GH	71.25 ± 20.25	90.71 ± 9.32	60.77 ± 16.31
validity, VT	81.50 ± 15.31	92.86 ± 2.67	75.38 ± 15.87
social function, SF	82.50 ± 7.48	85.71 ± 4.72	80.77 ± 8.25
role-emotion, RE	70.00 ± 10.26	76.19 ± 16.27	66.67 ± 0.00
mental health, MH	75.20 ± 16.75	85.14 ± 3.80	69.85 ± 18.66
Physical Component Summary, PCS	74.94 ± 12.99	86.54 ± 1.89	68.69 ± 12.02
Mental Component Summary, MCS	77.30 ± 9.51	84.98 ± 2.88	73.17 ± 9.29

**Table 3:** Narrative statistical analysis of daily activity.

Statistical Value	Average Value	Standard Deviation	Maximum	Minimum Value
Average daily standard deviation elliptical area (Km^2^)	9.77	13.40	59.2395	0.1512
Average daily moving distance (Km)	10.84	11.35	37.19	0.45
Average daily outing time (hours)	4.77	2.65	10.3	1.3

**Table 4:** Time-space path category data sheet.

Statistical Value	Average Value	Category (I)	Category (II)	Category (III)
PCS	74.94	62.58	78.88	80.78
MCS	77.30	70.85	76.76	81.38
Average daily standard deviation elliptical area (Km^2^)	9.77	0.94	4.25	17.27
Average daily moving distance (Km)	10.84	2.90	6.19	17.48
Average daily outing time (hours)	4.77	2.37	3.53	6.70

**Table 5:** Pearson correlation analysis table.

		PCS	MCS	Average Daily Standard Deviation Elliptical Area	Average Daily Moving Distance	Average Daily Outing Time
PCS	Pearson Correlation	1.000	0.897 **	0.480 *	0.369	0.450 *
	Sig. (two-tailed)		0.000	0.032	0.110	0.047
	N	20	20	20	20	20
MCS	Pearson Correlation	0.897 **	1.000	0.524 *	0.556 **	0.538 **
	Sig. (two-tailed)	0.000		0.018	0.011	0.014
	N	20	20	20	20	20
Average daily standard deviation elliptical area	Pearson Correlation	0.480 *	0.524 *	1.000	0.700 **	0.797 **
	Sig. (two-tailed)	0.032	0.018		0.001	0.000
	N	20	20	20	20	20
Average daily moving distance	Pearson Correlation	0.369	0.556 **	0.700 **	1.000	0.754 **
	Sig. (two-tailed)	0.110	0.011	0.001		0.000
	N	20	20	20	20	20
Average daily outing time	Pearson Correlation	0.450 *	0.538 **	0.797 **	0.754 **	1.000
	Sig. (two-tailed)	0.047	0.014	0.000	0.000	
	N	20	20	20	20	20

*. Correlation is significant at the 0.05 level (2-tailed). **. Correlation is significant at the 0.017 level (2-tailed).

**Table 6:** Pearson correlation analysis table.

		PCS	MCS	Age	Education Level
PCS	Pearson Correlation	1.000	0.897 *	0.353	−0.137
	Sig. (two-tailed)		0.000	0.127	0.564
	N	20	20	20	20
MCS	Pearson Correlation	0.897 *	1.000	0.251	−0.166
	Sig. (two-tailed)	0.000		0.286	0.483
	N	20	20	20	20
Age	Pearson Correlation	0.353	0.251	1.000	−0.619 *
	Sig. (two-tailed)	0.127	0.286		0.004
	N	20	20	20	20
Education level	Pearson Correlation	−0.137	−0.166	−0.619 *	1.000
	Sig. (two-tailed)	0.564	0.483	0.004	
	N	20	20	20	20

*. Correlation is significant at the 0.05 level (2-tailed).

**Table 7:** Group statistics table.

	Gender	N	Mean	Std. Dev.	Std. Err.
PCS	male female	7 13	86.71 68.85	1.890 12.020	0.714 3.334
MCS	male female	7 13	84.71 73.15	2.812 9.371	1.063 2.599

**Table 8:** Independent-samples *t*-test table.

		Levene’s Test for Equality of Variances		*t*-Test for Equality of Means						
		F	Sig.	T	df	Sig. (2-tailed)	Mean Difference	Std. Error Difference	95%Confidence Interval of the Difference	
									Lower	Upper
PCS	Equal variances assumed Equal variances not assumed	10.767	0.004	3.860 5.241	18 13.072	0.001 0.000	17.868 17.868	4.629 3.409	8.142 10.507	27.594 25.229
MCS	Equal variances assumed Equal variances not assumed	14.173	0.001	3.153 4.117	18 15.482	0.006 0.001	11.560 11.560	3.667 2.808	3.857 5.592	19.264 17.529

**Table 9:** Group statistics table.

Marital Status		N	Mean	Std. Dev.	Std. Err.
Average daily moving distance (km)	married widowed	16 4	12.686 3.478	11.953 3.249	0.2.988 1.624
Average daily outing time (hour)	married widowed	16 4	5.363 2.375	2.626 0.763	0.656 0.381
Average daily standard deviation elliptical area (Km^2^)	married widowed	16 4	11.718 3.4775	14.382 1.376	3.596 0.688

**Table 10:** Independent-samples *t*-test table.

		Levene’s Test for Equality of Variances		*t*-Test for Equality of Means						
		F	Sig.	T	df	Sig. (2-tailed)	Mean Difference	Std. Error Difference	95% Confidence Interval of the Difference	
									Lower	Upper
Average daily moving distance (km)	Equal variances assumed Equal variances not assumed	5.756	0.027	1.499 2.708	18 17.524	0.151 0.015	9.209 9.209	6.145 3.401	−3.700 2.049	22.118 16.368
Average daily outing time (hour)	Equal variances assumed Equal variances not assumed	3.458	0.079	2.211 3.935	18 17.092	0.040 0.001	2.988 2.988	1.351 0.759	0.149 1.386	5.826 4.589
Average daily standard deviation elliptical area (Km2)	Equal variances assumed Equal variances not assumed	2.001	0.174	1.328 2.665	18 16.012	0.201 0.017	9.758 9.758	7.346 3.661	−5.676 1.997	25.191 17.518

## Data Availability

Data available on request due to restrictions e.g., privacy or ethical.

## Supplementary Materials

The following are available online at https://www.mdpi.com/article/10.3390/ijerph18115902/s1, File S1: basic demographic characteristics data sheet, File S2: the short portable mental status QUESTIONNAIRE, File S3: the short-form-36 health survey (sf-36).
