# Supplementary material for: A Space-Time Analysis of Rural Older People’s Outdoor Mobility and Its Impact on Self-Rated Health: Evidence from a Taiwanese Rural Village
Source: Int J Environ Res Public Health. 2021 May 31;18(11):5902. doi: 10.3390/ijerph18115902 (PMC8198793; doi:10.3390/ijerph18115902)
Supplement: Supplementary file 1 [file ijerph-18-05902-s001.zip › ijerph-1188147-supplementary.pdf]

File S1: BASIC DEMOGRAPHIC CHARACTERISTICS DATA SHEET

A1. What is your gender?

□1. Male
□2. Female

A2. Which category below includes your age?

□1. 60 or younger
□2. 60-64
□3. 65 -69
□4. 70 - 74
□5. 75 -79
□6. 80 - 84
□7. 85 -89
□8. 90 -94
□9. 90 or older

A3. What is the highest level of school you have completed?

□1. Primary school
□2. junior middle school
□3. high school
□4. Colleges and Universities
□5. Above graduate school

A4. What is your occupation category?

□1. None
□2. Housewife
□3. Official
□4. Agriculture
□5. Industry
□6. Business
□7. Service industry
□8. General staff
□9. Other

A5. What religion do you believe in?

□1. No faith □2. Folk traditional belief □3. Buddhism
□4. Taoism
□5. Christianity
□6. Catholicism
□7. Islam
□8. Other

A6. **What is the source of your current average monthly living expenses? How much is it?**

□1. Work or business income □2. Provided by spouse or cohabitant
□3.Savings, interest, rent, investment income or commercial insurance payment
□4.Pension or social insurance
□5. Children or grandchildren (including daughter-in-law, son-in-law, daughter-in-law or son-in-law)
□6. Borrowing from others or financial institutions
□7. Government assistance or subsidy
□8. Social or family assistance
□9. Other

A7. **what is your marital status?**

□1. Never married
□2. Married
□3. Divorced
□4. Widowed
□5. Other

A8-1. **Please check your living environment and condition according to the type of residence.**

□1. Elevator building
□2. Apartment, is there an elevator?□①Yes □②No
□3. Is there an elevator in the house above two floors (including villa)?
□①Yes □②No
□4. Bungalow
□5. Shed

A8-2. **Who did you live with? in the past three months.**

□1. Living alone
□2. Living with others，Number of people living together：__________，Who are they？(Multiple choice)
□3. Parents (including spouse parents)
□4. Spouse (including cohabitant)
□5. Unmarried children
□6. Married children (including their spouses)
□7. Grandchildren
□8. Other relatives
□9. Friends
□10. Foreign nurses
□11. Other

A9-1. **Do you have private transportation? What is it?**

□1. None
□2. Bicycle
□3. Motorcycle
□4. Car
□5. Truck
□6. Electric vehicles
□7. Other

A9-2. **What kind of transportation mode do you usually use when you go out?**

□1. Walking
□2. Bicycle
□3. Motorcycle
□4. Car
□5. Truck
□6. Public transportation
□7. Electric vehicles
□8. Other

File S2: THE SHORT PORTABLE MENTAL STATUS QUESTIONNAIRE (SPMSQ)

Question	Response	Incorrect Responses
1. What are the date, month, and year?		
2. What is the day of the week?		
3. What is the name of this place?		
4. What is your phone number?		
5. How old are you?		
6. When were you born?		

7. Who is the current president?		
8. Who was the president before him?		
9. What was your mother's maiden name?		
10. Can you count backward from 20 by 3's?		

SCORING:*

0-2 errors: normal mental functioning
3-4 errors: mild cognitive impairment
5-7 errors: moderate cognitive impairment
8 or more errors: severe cognitive impairment
*One more error is allowed in the scoring if a patient has had a grade school education or less. *One less error is allowed if the patient has had education beyond the high school level.

Source: Pfeiffer, E. (1975). A short portable mental status questionnaire for the assessment of organic brain deficit in elderly patients. Journal of American Geriatrics Society. 23, 433-41.

File S3: THE SHORT-FORM-36 HEALTH SURVEY（SF-36）

Copyright c 1995 Health Assessment Lab. All rights reserved. (IQOLA SF-36 Taiwan Standard Version 1.0)

No: ______________ Date: _____________

This survey asks for your views about your health. This information will help keep track of how you feel and how well you are able to do your usual activities. Thank you for completing this survey!

F1. In general, would you say your health is:

□1. Excellent
□2.Very Good
□3.Good
□4.Fair
□5.Poor

F2. Compared to one year ago, how would you rate your health in general now?

□1. Much better now than one year ago
□2. Somewhat better now than one year ago
□3. About the same as one year ago
□4. Somewhat worse now than one year ago
□5. Much worse now than one year ago

F3. The following items are about activities you might do during a typical day. Does your health now limit you in these activities? If so, how much?

Activities	Yes, Limited A Lot	Yes, Limited A Little	No, Not Limited At All
a. Vigorous activities, such as running, lifting heavy objects, participating in strenuous sports	1	2	3
b. Moderate activities, such as moving a table, pushing a vacuum cleaner, bowling, or playing golf	1	2	3
c. Lifting or carrying groceries	1	2	3
d. Climbing several flights of stairs	1	2	3
e. Climbing one flight of stairs	1	2	3
f. Bending, kneeling, or stooping	1	2	3
g. Walking more than a mile	1	2	3
h. Walking several blocks	1	2	3
i. Walking one block	1	2	3
j. Bathing or dressing yourself	1	2	3

F4. During the past 4 weeks, have you had any of the following problems with your work or other regular daily activities as a result of your physical health?

	Yes	No
a. Cut down on the amount of time you spent on work or other activities	1	2
b. Accomplished less than you would like	1	2
c. Were limited in the kind of work or other activities	1	2
d. Had difficulty performing the work or other activities (for example, it took extra effort)	1	2

F5. During the past 4 weeks, have you had any of the following problems with your work or other regular activities as a result of any emotional problems (such as feeling depressed or anxious)?

	Yes	No
a. Cut down on the amount of time you spent on work or other activities	1	2

b. Accomplished less than you would like	1	2
c. Did work or other activities less carefully than usual	1	2

F6. During the past 4 weeks, to what extent has your physical health or emotional problems interfered with your normal social activities with family, friends, neighbors, or groups?

□1. Not at all
□2. A little bit
□3. Moderately
□4. Quite a bit
□5. Extremely

F7. During the past 4 weeks, to what extent has your physical health or emotional problems interfered with your normal social activities with family, friends, neighbors, or groups?

□1. None
□2. Very mild
□3. Mild
□4. Severe
□5. Very Severe

F8. During the past 4 weeks, how much did pain interfere with your normal work (including both work outside the home and housework)?

□1. Not at all
□2. A little bit
□3. Moderately
□4. Quite a bit
□5. Extremely

F9. These questions are about how you feel and how things have been with you during the past 4 weeks. For each question, please give the one answer that comes closest to the way you have been feeling. How much of the time during the past 4 weeks?

	All of the	Most of the Time	A Good Bit of the	Some of the Time	A Little of the Time	None of the Time

	Time		Time			
a. Did you feel full of pep?	1	2	3	4	5	6
b. Have you been a very nervous person?	1	2	3	4	5	6
c. Have you felt so down in the dumps that nothing could cheer you up?	1	2	3	4	5	6
d. Have you felt calm and peaceful?	1	2	3	4	5	6
e. Did you have a lot of energy?	1	2	3	4	5	6
f. Have you felt downhearted and blue?	1	2	3	4	5	6
g. Did you feel worn out?	1	2	3	4	5	6
h. Have you been a happy person?	1	2	3	4	5	6
i. Did you feel tired?	1	2	3	4	5	6

F10. During the past 4 weeks, how much of the time has your physical health or emotional problems interfered with your social activities (like visiting friends, relatives, etc.)?

□1. All of the time
□2. Most of the time
□3. Some of the time
□4. A little of the time
□5. None of the time

F11. How TRUE or FALSE is each of the following statements for you?

	Definitely True	Mostly True	Don't Know	Mostly False	Definitely False
a. I seem to get sick a little easier than other people	1	2	3	4	5
b. I am as healthy as anybody I know	1	2	3	4	5
c. I expect my health to get worse	1	2	3	4	5

d. My health is excellent	1	2	3	4	5
